# Evaluating Large Language Models for Sentiment Analysis and Hesitancy Analysis on Vaccine Posts From Social Media: Qualitative Study

**DOI:** 10.2196/64723

**Published:** 2025-10-15

**Authors:** Augustine Annan, Amanda L Eiden, Dong Wang, Jingcheng Du, Majid Rastegar-Mojarad, Varun Kumar Nomula, Xiaoyan Wang

**Affiliations:** 1 IMO Health, Rosemont, IL, United States; 2 Merck & Co, Inc, 126 East Lincoln Avenue, Rahway, NJ, 07065, United States, 1 267-305-0672

**Keywords:** vaccine sentiment, vaccine hesitancy, large language models, GPT4, social media platforms, public health communication, LLMs, NLP, machine learning, artificial intelligence, language models, sentiment analysis, hesitancy analysis, vaccine posts, social media, vaccine, public opinion, vaccine-related, public sentiment, computational efficiency

## Abstract

**Background:**

In the digital age, social media has become a crucial platform for public discourse on diverse health-related topics, including vaccines. Efficient sentiment analysis and hesitancy detection are essential for understanding public opinions and concerns. Large language models (LLMs) offer advanced capabilities for processing complex linguistic patterns, potentially providing valuable insights into vaccine-related discourse.

**Objective:**

This study aims to evaluate the performance of various LLMs in sentiment analysis and hesitancy detection related to vaccine discussions on social media and identify the most efficient, accurate, and cost-effective model for detecting vaccine-related public sentiment and hesitancy trends.

**Methods:**

We used several LLMs—generative pretrained transformer (GPT-3.5), GPT-4, Claude-3 Sonnet, and Llama 2—to process and classify complex linguistic data related to human papillomavirus; measles, mumps, and rubella; and vaccines overall from X (formerly known as Twitter), Reddit, and YouTube. The models were tested across different learning paradigms: zero-shot, 1-shot, and few-shot to determine their adaptability and learning efficiency with varying amounts of training data. We evaluated the models’ performance using accuracy, *F*_1_-score, precision, and recall. In addition, we conducted a cost analysis focused on token usage to assess the computational efficiency of each approach.

**Results:**

GPT-4 (*F*_1_-score=0.85 and accuracy=0.83) outperformed GPT-3.5, Llama 2, and Claude-3 Sonnet across various metrics, regardless of the sentiment type or learning paradigm. Few-shot learning did not significantly enhance performance compared with the zero-shot paradigm. Moreover, the increased computational costs and token usage associated with few-shot learning did not justify its application, given the marginal improvement in model performance. The analysis highlighted challenges in classifying neutral sentiments and convenience, correctly interpreting sarcasm, and accurately identifying indirect expressions of vaccine hesitancy, emphasizing the need for model refinement.

**Conclusions:**

GPT-4 emerged as the most accurate model, excelling in sentiment and hesitancy analysis. Performance differences between learning paradigms were minimal, making zero-shot learning preferable for its balance of accuracy and computational efficiency. However, the zero-shot GPT-4 model is not the most cost-effective compared with traditional machine learning. A hybrid approach, using LLMs for initial annotation and traditional models for training, could optimize cost and performance. Despite reliance on specific LLM versions and a limited focus on certain vaccine types and platforms, our findings underscore the capabilities and limitations of LLMs in vaccine sentiment and hesitancy analysis, highlighting the need for ongoing evaluation and adaptation in public health communication strategies.

## Introduction

In the era of digital communication, social media platforms have become central to the dissemination and exchange of public opinions on health-related topics, including vaccination. The vast and dynamic nature of these platforms offers a rich dataset for analyzing public sentiment and hesitancy toward vaccines, which is critical for developing effective health communication strategies. The advent of advanced artificial intelligence (AI) technologies, particularly large language models (LLMs) such as OpenAI’s generative pretrained transformer (GPT-4.0) [1] and GPT-3.5 [2], Anthropic’s Claude 3 Sonnet [3], and Meta’s Llama 2 [4], represents a significant leap forward in our capacity to understand and interpret extensive amounts of unstructured text data [5]. These models have achieved impressive results in a variety of applications; for example, they significantly aid in diagnostics and personalizing treatment approaches and prove successful in medical licensing examinations and specialized medical tasks [6-12], thereby showcasing the expansive range of their capabilities [13-15].

The use of LLMs extends beyond traditional medical applications. These technologies are set to transform public health research by offering insights into public sentiments expressed on the web, particularly on complex topics such as vaccine hesitancy; defined by reluctance or delay in accepting vaccines despite the availability of vaccination services [16], vaccine hesitancy is a barrier to global health initiatives, especially in ongoing campaigns against diseases such as human papillomavirus (HPV) and measles, mumps, and rubella (MMR). Addressing this challenge requires a nuanced understanding of public opinions, concerns, and misinformation patterns, which LLMs are uniquely positioned to facilitate [17].

The rapid evolution of LLMs, from earlier models such as GPT-2 and BERT to more advanced iterations, has brought substantial improvements in language comprehension and task-solving abilities [1,2,18-20]. Other organizations, including Anthropic with its Claude models [3,21] and Meta AI with the Llama series [4,22], have also contributed significantly to this field.

Despite these advancements, research comparing different LLMs and learning paradigms in public health sentiment analysis remains limited. This study aims to address this gap by evaluating the efficacy of various LLMs in analyzing sentiments and hesitancy related to HPV, MMR, and general vaccines across 3 social media platforms: X (formerly Twitter), Reddit, and YouTube.

In this work, we explored the adaptability and effectiveness of LLMs in processing health-related sentiments by using zero-shot, 1-shot, and few-shot learning paradigms. A statistical comparison of performance across these paradigms reveals significant insights into optimizing model efficiency and resource allocation for large-scale sentiment analysis. Specifically, our analysis delves into the practical implications of selecting learning paradigms based on their computational costs and accuracy in detecting nuanced expressions of vaccine sentiment and hesitancy.

By pinpointing the optimal combinations of LLMs and learning paradigms for robust sentiment and hesitancy analysis, our research directly informs the strategic deployment of AI in crafting targeted public health messaging. This endeavor not only enhances our understanding of public discourse around vaccines but also provides a foundation for addressing vaccine hesitancy more effectively, thereby contributing to the advancement of public health communication strategies.

## Methods

### Data Collection and Annotation

We analyzed English language social media posts from X (formerly Twitter), Reddit, and YouTube, focusing on public sentiment toward general vaccines, HPV, and MMR vaccines. Data collection spanned from January 1, 2011, to October 31, 2021. We selected these platforms because they provide robust application programming interface (API) access and represent a significant share of vaccine-related discourse on the web. English language posts were prioritized due to their widespread usage in large markets and accessibility. The detailed data collection and annotation procedures follow the methodology described in a previous study [23]. Data were retrieved using the APIs provided by the platforms, following their data privacy and ethical guidelines.

To ensure the relevance and quality of the data, we tailored our search queries for each platform, as variations in text format and query logic required customization. Inclusion keywords such as “vaccine,” “immunization,” “HPV vaccine,” and “MMR vaccine” were used, while exclusion keywords (eg, “software updates” or “sports immunities”) filtered out irrelevant content. Duplicate posts and spam were removed during preprocessing.

The data collection period includes the COVID-19 pandemic, a significant global event that likely influenced public sentiment and hesitancy discussions on vaccines. With the widespread availability of COVID-19 vaccines during this time, public discourse around vaccines may have become more polarized, amplifying safety concerns and misinformation. Although this study does not focus on COVID-19–specific sentiments, the pandemic era context may have shaped the overall tone and topics of vaccine-related discussions. Posts unrelated to COVID-19 vaccines were also included to capture general vaccine sentiment trends, ensuring that the analysis remained relevant to MMR, HPV, and general vaccines.

Our study involved a dual-layered annotation approach for 10,485 social media posts. Posts were first categorized for sentiment (positive, neutral, or negative) and then for vaccine hesitancy. The hesitancy annotation involved a 2-step process: initially determining whether a post was hesitant or nonhesitant, and for those identified as hesitant, further categorizing them based on the World Health Organization (WHO’s 3Cs [confidence, complacency, and convenience] model) [24].

To ensure balance, we evenly annotated 1165 posts across 3 platforms (X, Reddit, and YouTube) and vaccine types (general, HPV, and MMR). The final dataset comprised 36% positive, 26% neutral, and 38% negative sentiments, with 39% of posts identified as hesitant. Among hesitant posts, 58% reflected confidence issues, 25% complacency, and 17% convenience concerns.

Posts were annotated for sentiment as positive (favorable opinions or experiences regarding vaccines), neutral (informational or mixed sentiment posts), or negative (adverse opinions or potentially deterring information). Hesitancy was annotated based on the WHO’s 3Cs model: confidence (distrust in vaccine efficacy, safety, or delivery systems), complacency (low perceived risk of vaccine-preventable diseases), and convenience (barriers to vaccine accessibility or difficulties in obtaining vaccination) [23].

### Few-Shot Learning and Evaluation

For evaluation, 7515 annotated posts were used to assess the LLMs’ performance. Table 1 provides the distribution of these posts across sentiment and hesitancy categories, proportional to the overall annotated dataset. A detailed distribution of annotated posts in each sentiment and 3Cs construct for each platform and vaccine topic group is shown in Table S1 in Multimedia Appendix 1.

**Table 1.** Distribution of evaluation posts by sentiment and hesitancy categories.

Category	Count of posts	Percentage
Sentiment		
Positive	2705	36
Neutral	1954	26
Negative	2856	38
Hesitancy		
Nonhesitant	4584	61
Hesitant	2931	39
Confidence	1850	63% of hesitant
Complacency	1020	35% of hesitant
Convenience	720	25% of hesitant

Few-shot learning experiments were conducted separately for each vaccine type and each platform. For each experiment, posts were selected using a balanced sampling approach to ensure balanced representation across sentiment categories (positive, neutral, and negative). For each configuration (eg, 5-shot), we sampled an equal number of examples per sentiment category. Specifically, for 5-shot learning, we included 5 examples from each sentiment category (positive, neutral, and negative) per vaccine type per platform. This resulted in a total of 15 examples per vaccine type per platform for 5-shot learning. The same sampling approach was applied to other configurations, ensuring consistency and diversity in training examples across platforms and vaccine topics.

A similar methodology was used for hesitancy analysis. Posts were sampled to represent hesitant and nonhesitant categories as well as the WHO’s 3Cs constructs (confidence, complacency, and convenience), ensuring balanced representation across platforms and vaccine types.

To maintain consistency and enable fair comparisons, the same few-shot examples were reused across experiments for a given configuration, minimizing variability in training data and ensuring a controlled evaluation of model performance.

#### Sentiment Annotation

Sentiment annotation was a multiclass classification task, assigning 1 of 3 sentiment labels to each post:

- Positive: Posts that mention, report, or share positive opinions, news, or experiences about vaccines or vaccination.
  - *Example*: “Getting vaccinated is a vital step to safeguard your health and future.”
- Neutral: Posts that relate to vaccines or vaccination topics but contain no clear sentiment, express mixed sentiments, or do not explicitly advocate for or against vaccination.
  - *Example*: “HPV has multiple strains, and vaccines cover some of them but not all.”
- Negative: Posts that mention, report, or share negative opinions, news, or experiences about vaccines or vaccination, which may discourage vaccination.
  - *Example*: “The government is pushing vaccines despite the growing number of adverse reactions.”

#### 3Cs Vaccine Hesitancy Annotation

The annotation of vaccine hesitancy was based on the WHO’s 3Cs model, with each construct evaluated independently. Posts labeled as “Lack of confidence,” “Complacent,” or “Inconvenient” were considered hesitant, while posts without any of these constructs were labeled as nonhesitant. Definitions and an example for each construct are as follows:

- Lack of confidence: Posts reflecting mistrust in vaccine efficacy, safety, vaccine delivery system, or motivations of policy makers.
  - *Example*: “Vaccinated individuals are still catching the disease, so how effective are these shots really?”
- Complacency: Posts where the perceived risks of vaccine-preventable diseases are low and vaccination is deemed unnecessary.
  - *Example*: “I never got vaccinated as a kid and I turned out to be fine, so vaccines aren’t critical or essential.”
- Inconvenience: Posts highlighting physical, geographical, financial, or systemic barriers to vaccination, including issues related to health literacy and service accessibility.
  - *Example*: “I wanted to get the vaccine, but the nearest clinic is three hours away, and I cannot take time off work.”

The annotation was performed by trained annotators with a medical background. All annotators underwent training with a guideline developed for this study. Annotators achieved a high interannotator agreement score (Cohen κ=0.93), indicating strong reliability. Discrepancies were resolved through discussion and consensus. Examples of annotated posts and the full annotation framework are detailed in a previous paper [23].

### Implementation of LLMs

In this study, we leveraged 4 advanced LLMs: OpenAI’s GPT-3.5 and GPT-4, Anthropic’s Claude 3 Sonnet, and Meta’s Llama 2. These models were chosen for their effectiveness in natural language processing (NLP) tasks such as text generation, sentiment analysis, and question answering, making them ideal for analyzing sentiments and hesitancy in social media discourse about vaccination.

GPT-3.5, with its 175 billion parameters, offers substantial text generation and understanding capabilities [25]. GPT-4 builds on this foundation with enhanced nuanced text interpretation and more sophisticated training techniques, making it particularly effective for detailed analysis of complex topics such as vaccine sentiment in social media conversations [26]. Claude 3 Sonnet was selected for its advanced processing capabilities, improved steerability, and the ability to handle up to 200,000 tokens, making it particularly suitable for comprehensive sentiment analysis [27]. Meta’s Llama 2 was chosen for its open-source nature, flexibility, and robust performance in detecting nuanced sentiments and hesitancies, providing a strategic advantage for academic research.

### Model Evaluation Through Learning Paradigms

We evaluated the LLMs using 3 learning paradigms—zero-shot, 1-shot, and few-shot learning—to test their adaptability and efficiency with varying amounts of prior information, simulating real-world scenarios where labeled data may be scarce.

#### Zero-Shot Learning

In the zero-shot learning paradigm, the model analyzes tasks without any prior examples or specific training related to the task [28]. It relies entirely on its pretrained knowledge to infer the correct output. For our study, models classified vaccine sentiment or hesitancy from social media posts using only a carefully crafted prompt, without any sample posts or classifications. This setup tests the model’s ability to generalize from unrelated training data to new, unseen tasks.

#### One-Shot Learning

In 1-shot learning, the model is provided with a single example post for each category before making predictions. This minimal context helps the model understand the task with just 1 reference point per sentiment class or hesitancy construct.

#### Few-Shot Learning

Few-shot learning involves presenting the model with a set of multiple examples (*k* examples) to enhance its understanding and classification accuracy [29,30]. This methodology includes variations such as 5-shot and 10-shot settings. For sentiment analysis, *k* examples were provided for each category (positive, neutral, and negative). For hesitancy analysis, *k* examples were provided for each of the WHO’s 3Cs model dimensions (confidence, complacency, and convenience). The few-shot learning approach allowed us to explore how models perform with an increasing number of examples, testing their precision in detecting and classifying sentiments and hesitancies with minimal and slightly more information.

This structured approach confirms the flexibility and effectiveness of the models in adapting to different levels of available information. It provides a comprehensive perspective on their use in analyzing a broad spectrum of public opinions. By evaluating these models in scenarios that mirror real-world data availability, we aim to demonstrate the potential of LLMs to swiftly and accurately analyze complex vaccine-related social media discourse, potentially supporting enhanced public health communication strategies.

### Prompt Schema Design

The prompts used in this study were carefully crafted to align with the definitions and instructions provided to human annotators for vaccine sentiment and hesitancy terms. This approach, known as prompt engineering [31], ensured that the models’ interpretation matched human understanding, thereby enhancing prediction relevance and accuracy.

**Figure 1.**
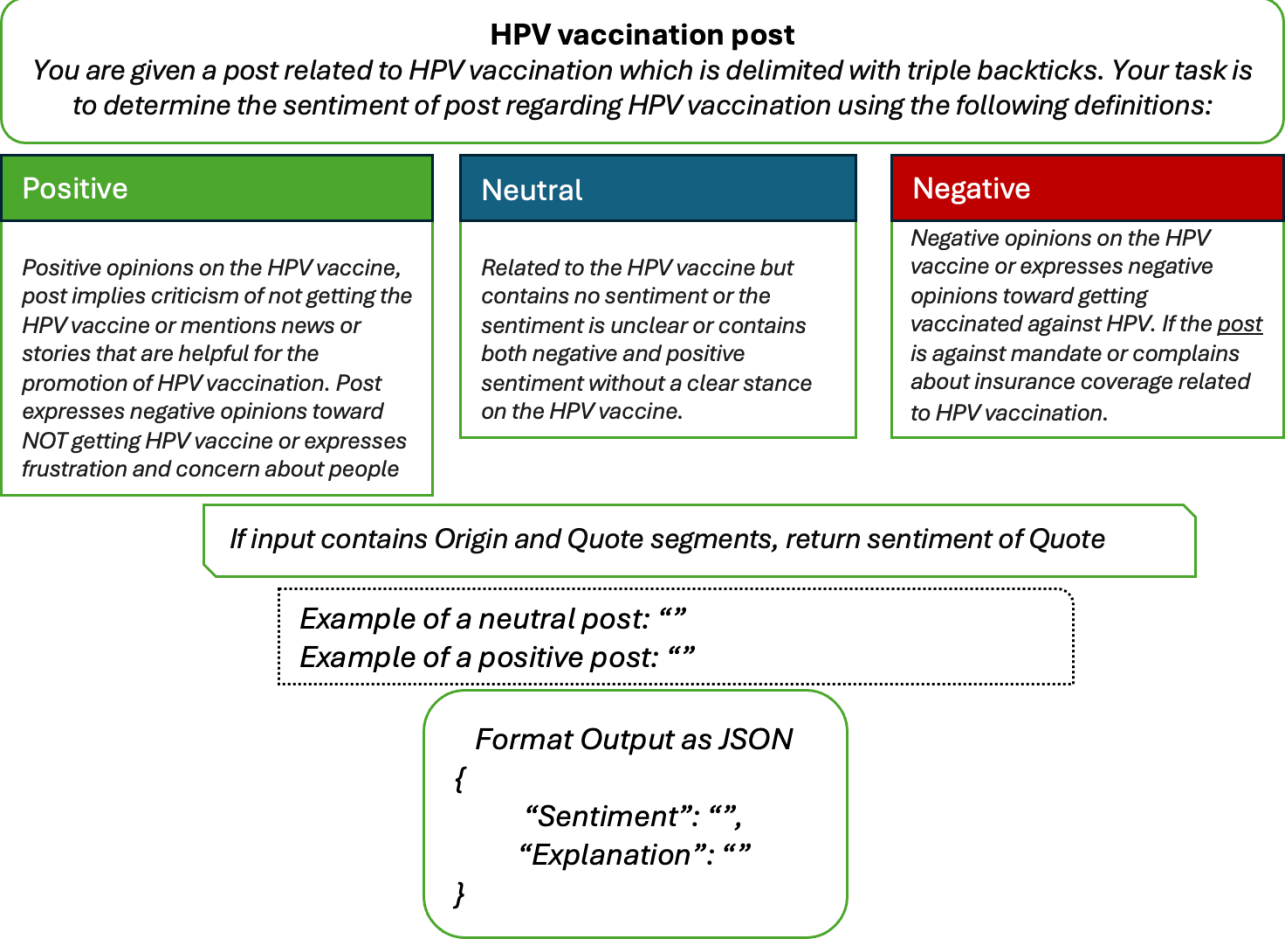
Prompt example for sentiment analysis. The text inside the dashed box is demonstrations of the few-shot setting and would be removed under the zero-shot setting. HPV: human papillomavirus; JSON: JavaScript Object Notation.

Prompts included explicit definitions and criteria for categorizing sentiments (eg, “positive,” “neutral,” and “negative”) and hesitancy constructs based on the WHO’s 3Cs model (confidence, complacency, and convenience) [15]. For example, in analyzing HPV vaccination–related posts, the prompt specified that quoted content should take precedence in cases of mixed sentiment and provided guidance for handling ambiguous language (Figure 1). Prompts also included requests for the models to provide explanations for their classifications, which enhanced interpretability and supported iterative refinement during the development phase [32].

Preliminary testing compared multiple prompt versions to evaluate clarity, task alignment, and interpretability. For instance, variations in linguistic framing, the level of detail provided, and the structure of schema-specific instructions were tested using a subset of the dataset. Prompts that introduced ambiguity or failed to consistently elicit accurate classifications were refined or excluded. The final schema was selected based on its ability to achieve robust and consistent performance across platforms, vaccine types, and sentiment categories.

The final schema used in this study has been disclosed in the supplementary material for transparency and reproducibility (Multimedia Appendix 2). Future research could explore the impact of alternative prompt designs, including variations in linguistic framing, level of detail, and schema-specific instructions, on model performance. Such studies would be particularly valuable for tasks requiring the interpretation of complex or ambiguous language.

By incorporating these detailed and context-specific instructions into our prompts, we ensured that the LLMs were well equipped to interpret and analyze the sentiment of vaccine-related social media posts accurately [33]. This approach underscores the critical role of prompt engineering in leveraging the full capabilities of LLMs for nuanced tasks such as sentiment analysis, where the subtleties of language and expression necessitate a high degree of precision and clarity in the instructions provided to the models.

### Hyperparameter Settings

We predefined 2 key hyperparameters to optimize the LLMs’ performance: temperature and maximum tokens. The temperature parameter, which controls output randomness, was set to zero to ensure deterministic responses. This setting is crucial in the context of health-related sentiment analysis, where consistency and reproducibility are paramount. The maximum token (words and subwords) limit was set to 512, allowing models to provide detailed reasoning for their classifications, which is valuable for understanding the nuances of vaccine-related sentiments.

To ensure reliability, each social media post was processed through each LLM 3 times. We achieved a 97% consistency rate in categorizations across iterations. For the 3% of inconsistent cases, we used a majority vote approach to determine the final category. This rigorous process enhances the credibility of our results in the context of public health communication research.

### Evaluation Metrics

To assess the effectiveness of the LLMs, within the framework of our study, we relied on a suite of established NLP evaluation metrics: accuracy, precision, recall, and the *F*_1_-score [34]. These metrics collectively offer a comprehensive snapshot of the models’ performance across various learning paradigms, ensuring a well-rounded assessment of their capabilities. These metrics were calculated for each sentiment and hesitancy category. Moreover, to ensure the understanding of the models’ performance, these evaluations were conducted separately for each vaccine type under discussion and across the various social media platforms included in our study.

### Statistical Analysis for Model Comparison

To rigorously compare LLM performance across zero-shot, 1-shot, and few-shot learning paradigms, we conducted ANOVA tests [35]. We focused on *F*_1_-scores and accuracy as key performance indicators. This analysis helps determine the most effective learning approach for sentiment and hesitancy analysis in vaccine-related social media discussions, a critical aspect of modern public health monitoring.

We set the significance level at *P*<.05 for identifying statistically significant differences. All statistical analyses were performed using R (version 4.2.2; R Core Team) providing a rigorous and replicable framework for our evaluations. This comprehensive evaluation framework allows us to assess the scalability and applicability of these AI models in real-world public health scenarios, potentially informing strategies for efficient, large-scale analysis of vaccine sentiments and hesitancy in diverse digital environments.

### Ethical Considerations

All data used in this study were publicly available, and ethical guidelines for social media research were followed. Data privacy policies of the platforms (X, Reddit, and YouTube) were adhered to, and all posts were deidentified using unique random IDs. To further safeguard privacy, no actual posts are directly quoted or reproduced in this manuscript. Instead, illustrative examples provided in the text are entirely theoretical and were crafted to reflect the general themes and patterns observed in the data. These examples ensure that no identifiable user information is disclosed, while still providing clarity on the study’s methodology and findings.

## Results

### Sentiment Analysis Overview

This section provides an overview of the sentiment analysis results, comparing the performance of the LLMs based on accuracy, *F*_1_-score, precision, and recall. We focused on the models’ abilities to classify sentiments (positive, neutral, and negative) in a zero-shot learning setting (Table 2).

**Table 2.** Large language models' performance (measured in accuracy, recall, precision, and *F*_1_-score) on vaccine sentiment analysis.

Model	Sentiment	Accuracy (%)	*F*_1_-score (%)	Precision (%)	Recall (%)
GPT-3.5	Negative	0.801	0.753	0.672	0.834
GPT-3.5	Neutral	0.821	0.633	0.612	0.659
GPT-3.5	Positive	0.819	0.781	0.711	0.861
GPT-4	Negative	0.940	0.932	0.910	0.950
GPT-4	Neutral	0.932	0.901	0.891	0.910
GPT-4	Positive	0.921	0.930	0.920	0.940
Claude-3s	Negative	0.882	0.801	0.841	0.760
Claude-3s	Neutral	0.821	0.690	0.701	0.681
Claude-3s	Positive	0.870	0.820	0.780	0.862
Llama 2	Negative	0.749	0.641	0.691	0.590
Llama 2	Neutral	0.719	0.140	0.110	0.221
Llama 2	Positive	0.721	0.421	0.411	0.418

GPT-4 exhibited the highest performance across all sentiment types, with accuracy ranging from 92% to 94%, *F*_1_-scores from 90% to 93%, precision from 89% to 92%, and recall from 91% to 95%. Claude-3 followed, with accuracy between 82% and 88%, *F*_1_-scores from 69% to 82%, precision from 70% to 84%, and recall from 68% to 86%. GPT-3.5 showed comparable performance with Claude-3, with accuracy ranging from 80% to 82%, *F*_1_-scores from 63% to 78%, precision from 61% to 71%, and recall from 66% to 86%. Llama 2 exhibited the lowest performance, with accuracy between 72% and 75%, *F*_1_-scores from 14% to 64%, precision from 11% to 69%, and recall from 22% to 59%.

### Insights From Shot-Learning Paradigms

**Figure 2.**
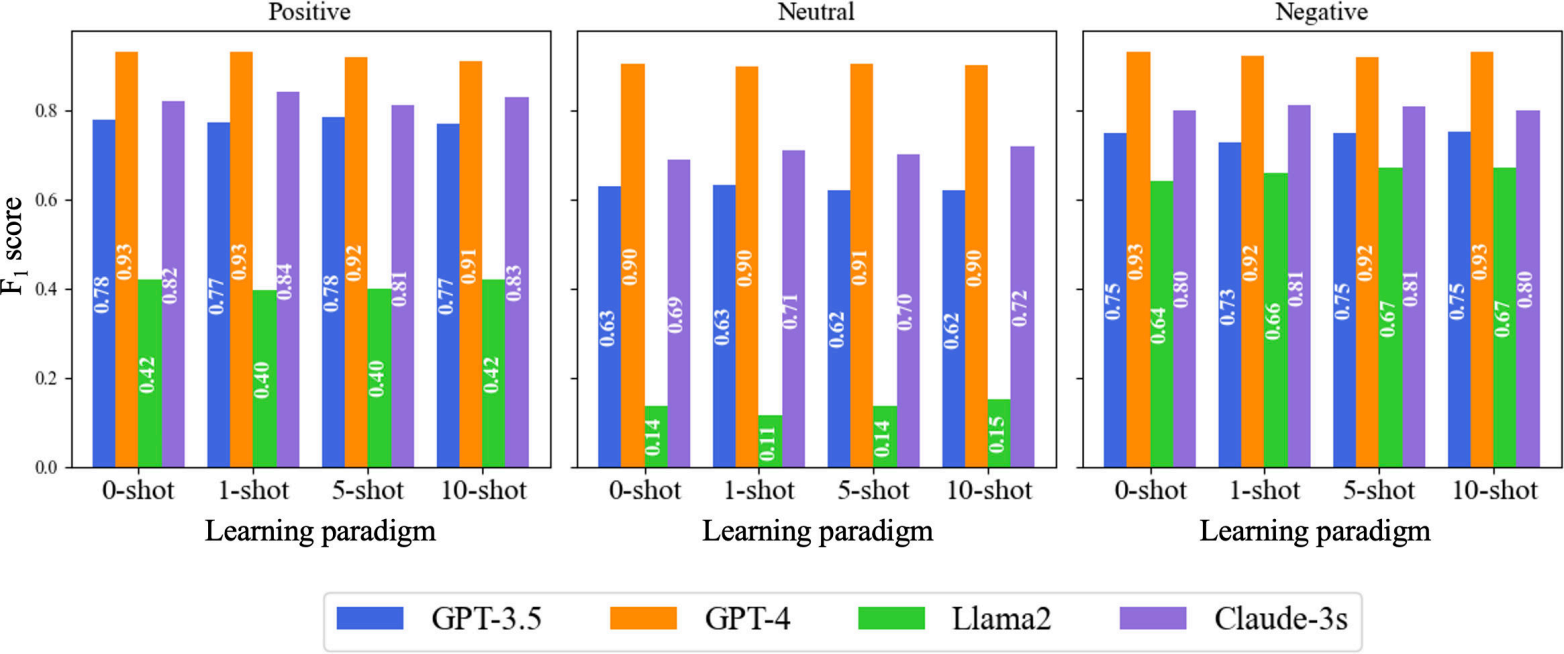
*F*_1_-score performance of large language models in sentiment analysis across learning paradigms.

We evaluated the performance of the 4 LLMs across zero-shot, 1-shot, 5-shot, and 10-shot learning paradigms. The results represent the averaged performance across vaccine types (HPV, MMR, and general) and platforms (X, Reddit, and YouTube), ensuring a comprehensive evaluation. The models’ accuracy and *F*_1_-scores remained relatively consistent across different paradigms for each sentiment type (Figures2  3). This consistency suggests that the models can effectively learn and generalize from few examples, maintaining their relative performance.

**Figure 3.**
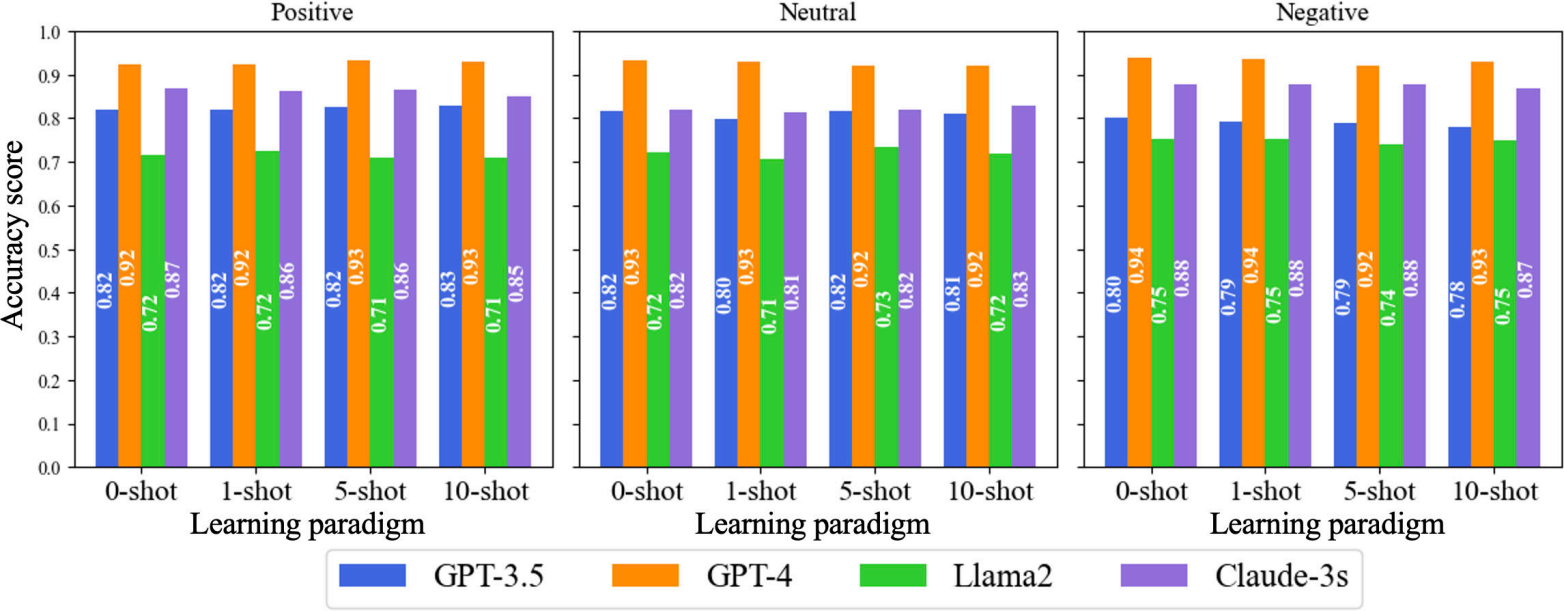
Accuracy performance of large language models in sentiment analysis across learning paradigms.

For GPT-4, accuracy scores for positive sentiment started at 92% in the zero-shot setting and increased marginally to 93.0% in the 10-shot setting. Neutral sentiment accuracy remained stable, ranging between 92.0% and 93%, while for negative sentiment, accuracy improved slightly from 92.0% in the 5-shot setting to 93.0% in the 10-shot setting. *F*_1_-scores for GPT-4 reflected similar stability, with the highest values observed for negative sentiment, reaching 93% in both zero-shot and 10-shot settings.

GPT-3.5 demonstrated stable performance across paradigms. Accuracy for positive sentiment rose slightly from 82% in the zero-shot setting to 83% in the 10-shot setting, while neutral sentiment accuracy fluctuated around 80%. Negative sentiment accuracy, however, decreased from 80% in the zero-shot setting to 78% in the 10-shot setting. *F*_1_-scores followed a similar pattern, with positive sentiment improving to 78% in the 5-shot setting before slightly declining to 77% in the 10-shot setting.

Claude-3s showed consistent performance across paradigms. Accuracy for neutral sentiment increased from 82% in the zero-shot setting to 83% in the 10-shot setting, while for positive sentiment, accuracy decreased slightly from 87% in the zero-shot setting to 85% in the 10-shot setting. *F*_1_-scores for negative sentiment remained stable across all paradigms, with values around 80%.

Llama 2 exhibited modest improvements with additional examples. Accuracy for positive sentiment improved slightly from 71% in the zero-shot setting to 72% in the 1-shot setting, stabilizing at 71% in the 5- and 10-shot settings. *F*_1_-scores for neutral sentiment remained low, reaching a maximum of 15% in the 10-shot setting. However, for negative sentiment, *F*_1_-scores improved from 64% in the zero-shot setting to 67% in the 5- and 10-shot settings.

Overall, the results highlight GPT-4’s superior performance in sentiment analysis across all paradigms, with consistent improvements observed as the number of examples increased. While GPT-3.5 and Claude-3s also exhibited stable and moderate performance, Llama2 showed the greatest need for further optimization despite modest gains in certain settings.

### Performance Across Vaccine Types and Social Media Platforms in Zero-Shot Settings

**Figure 4.**
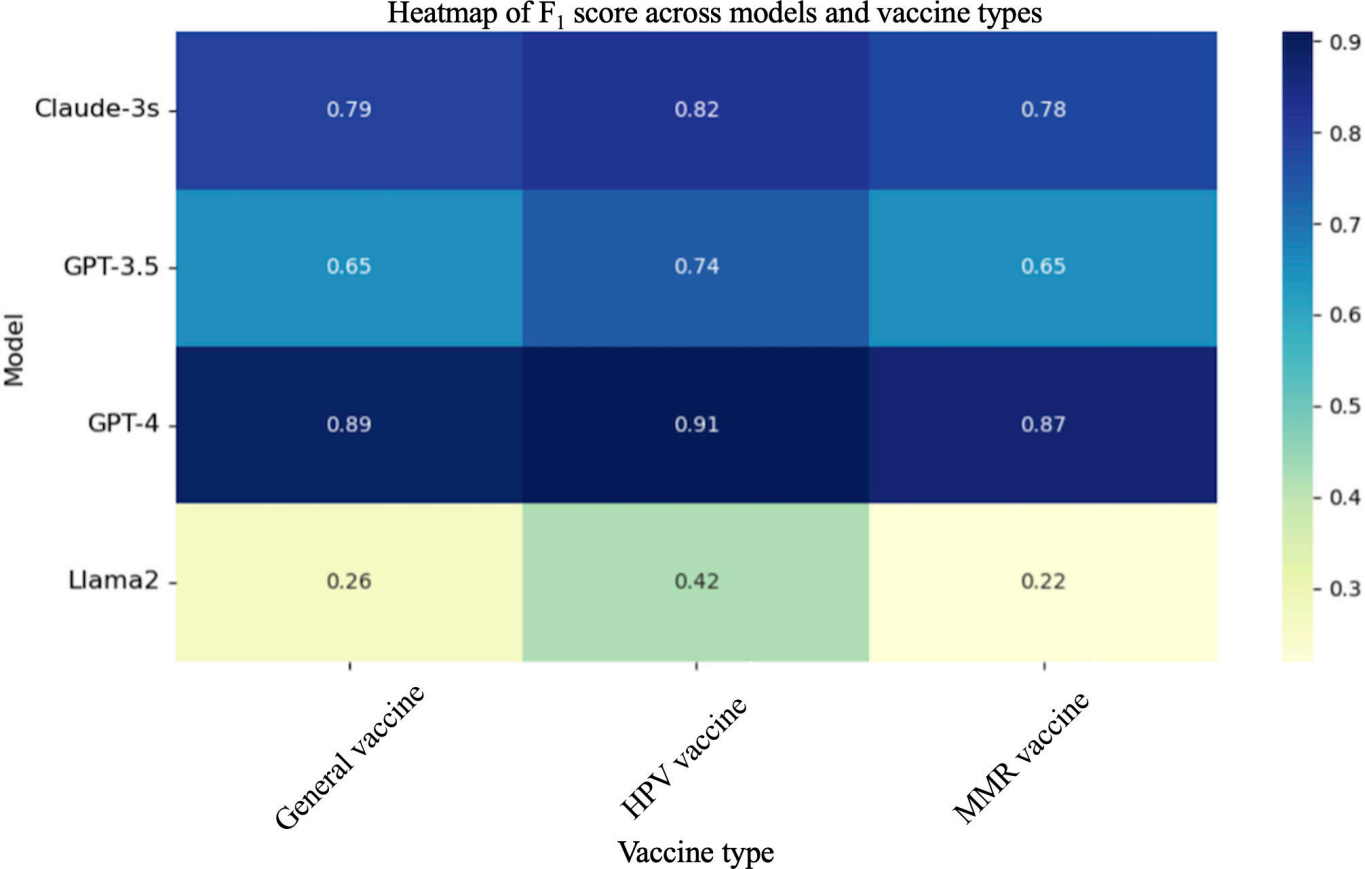
Zero-shot *F*_1_-scores across models and vaccine types. HPV: human papillomavirus; MMR: measles, mumps, and rubella.

GPT-4 demonstrated the highest *F*_1_-scores, ranging from 87% to 91%, showcasing its strong capability to generalize and accurately classify sentiment across all vaccine categories. Claude-3 followed closely with *F*_1_-scores between 78% and 82%, indicating its robust performance in the zero-shot setting. GPT-3.5 showed moderate performance with *F*_1_-scores ranging from 65% to 85%. Llama 2 had the lowest scores, between 22% and 42%, highlighting the variability in performance among the models (Figure 4).

Furthermore, we present the performance of the 4 language models in sentiment analysis across 3 social media platforms: Reddit, X, and YouTube (Table 2). While the overall performance trend remains consistent across platforms, there are minor variations worth noting. GPT-3.5 exhibited a slightly higher *F*_1_-score on YouTube (71.9%) than Reddit (71.4%) and X (70.6%). Claude-3 showed a higher *F*_1_-score on YouTube (78.9%) than X (78.6%) and Reddit (77.5%). Llama 2 had a higher *F*_1_-score on YouTube (41.5%) than X (39.9%) and Reddit (38.8%). GPT-4 maintained consistent performance across platforms with *F*_1_-scores ranging from 91.7% to 91.8%.

### Hesitancy Analysis Overview

GPT-4 led in both sentiment and hesitancy analyses, although its accuracy scores in hesitancy analysis (83%-96%) were slightly lower than in sentiment analysis (92%-94%; Table 3). This suggests that accurately identifying and classifying hesitancy types might be marginally more challenging than sentiment analysis, even for the best-performing model (Table 4). Claude-3 showed consistent performance across both tasks, with accuracy scores in hesitancy analysis (80%-87%) comparable with sentiment analysis (82%-88%). GPT-3.5 and Llama 2 exhibited slight improvements in accuracy for some hesitancy types compared with their sentiment analysis performance. For instance, GPT-3.5 achieved an accuracy of 84.9% for convenience hesitancy, higher than its sentiment analysis accuracy scores (80%-82%), while Llama 2’s accuracy for confidence hesitancy (70%) was close to its performance in negative sentiment analysis (75%).

**Table 3.** Large language models' performance (measured in accuracy and *F*_1_-score) across social media platforms on vaccine sentiment analysis.

Metric	Reddit				X, formerly known as Twitter				YouTube			
	GPT-3.5	GPT-4	Claude3s	Llama2	GPT-3.5	GPT-4	Claude3s	Llama2	GPT-3.5	GPT-4	Claude3s	Llama2
Accuracy	0.809	0.929	0.852	0.731	0.809	0.929	0.851	0.729	0.81	0.928	0.854	0.722
*F*_1_-score	0.714	0.917	0.775	0.388	0.706	0.918	0.786	0.399	0.719	0.918	0.789	0.415

**Table 4.** Large Language models' performance (measured in accuracy, recall, precision, and *F*_1_-score) on vaccine hesitancy analysis.

Metric and hesitancy type	GPT-3.5	GPT-4	Claude 3s	Llama 2
Accuracy				
Confidence	0.789	0.832	0.801	0.700
Convenience	0.849	0.961	0.871	0.729
Complacency	0.823	0.864	0.844	0.699
Hesitancy	0.762	0.878	0.844	0.668
Precision				
Confidence	0.855	0.957	0.796	0.614
Convenience	0.764	0.849	0.819	0.532
Complacency	0.621	0.696	0.679	0.286
Hesitancy	0.755	0.851	0.811	0.548
Recall				
Confidence	0.679	0.759	0.849	0.575
Convenience	0.799	0.80	0.790	0.492
Complacency	0.784	0.867	0.897	0.135
Hesitancy	0.677	0.856	0.849	0.479
*F*_1_-score				
Confidence	0.757	0.847	0.757	0.594
Convenience	0.781	0.824	0.804	0.511
Complacency	0.693	0.772	0.773	0.183
Hesitancy	0.714	0.853	0.823	0.511

### Impact of Learning Paradigms on Hesitancy Analysis

**Figure 5.**
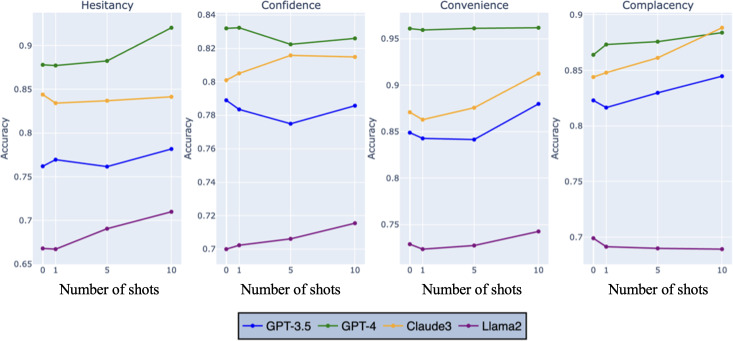
Model accuracy performance by hesitancy type and number of shots.

We explored the impact of zero-shot, 1-shot, 5-shot, and 10-shot learning paradigms on the models’ hesitancy analysis capabilities, focusing on accuracy and *F*_1_-scores. The results indicate that GPT-4 continued to excel, with accuracy scores ranging from 83.2% for confidence in the zero-shot setting to 96% for convenience in the zero-shot setting (Figure 5). Notably, GPT-4’s performance improves as the number of shots increases, particularly for overall hesitancy, reaching an accuracy of 92% in the 10-shot setting. This showcases GPT-4’s exceptional ability to learn from additional examples and accurately detect overall vaccine hesitancy.

**Figure 6.**
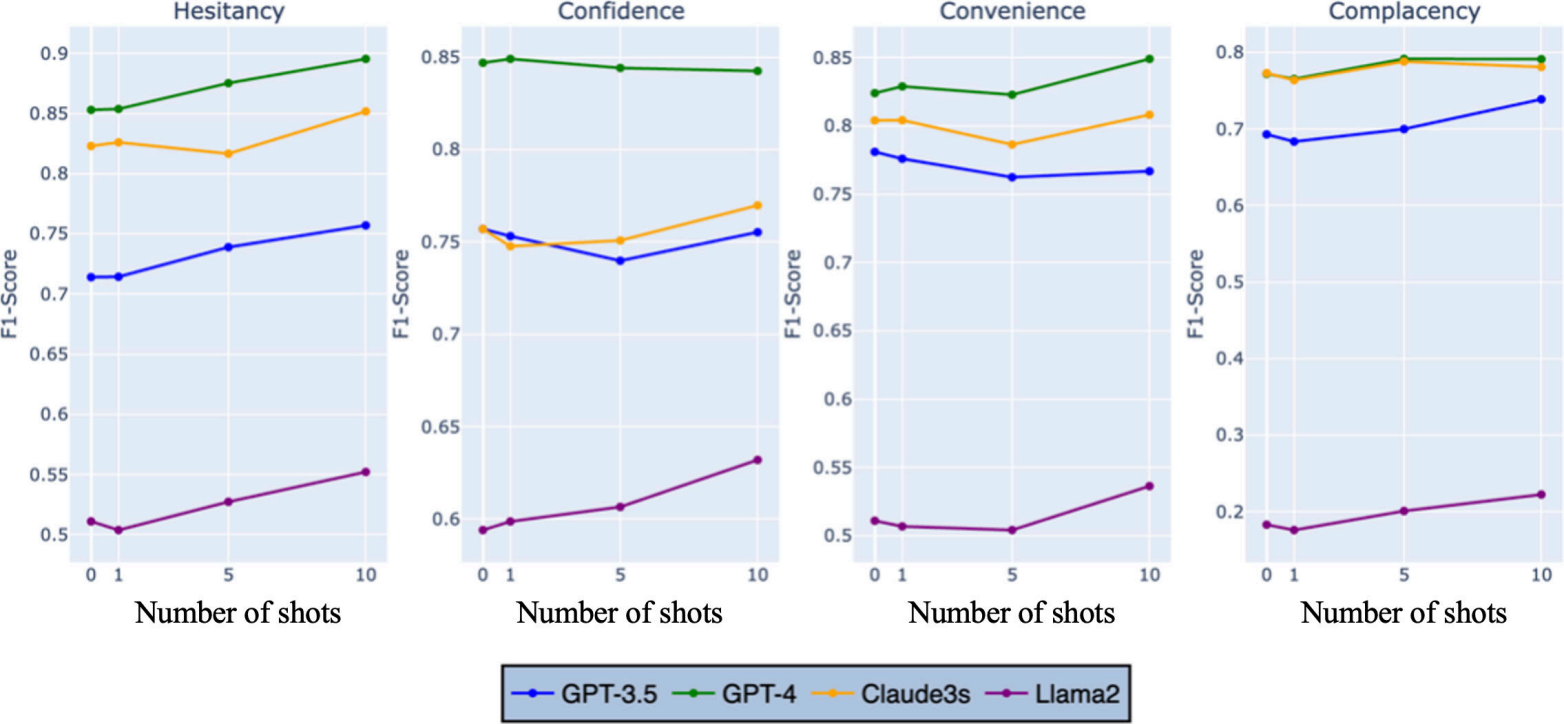
Model *F*_1_-score performance by hesitancy type and number of shots.

The *F*_1_-scores tell a similar story. GPT-4 maintained its lead, with *F*_1_-scores ranging from 77.2% for complacency in the zero-shot setting to 89.5% for overall hesitancy in the 10-shot setting (Figure 6). The increase in *F*_1_-scores as the number of shots grows highlights GPT-4’s ability to not only accurately classify hesitancy but also comprehensively identify relevant instances.

Claude-3 showed substantial improvement in accuracy for convenience hesitancy as the number of shots increased, reaching 91.2% in the 10-shot setting. This underscores Claude-3’s adaptability and potential to capture specific aspects of hesitancy when provided with more examples. GPT-3.5 exhibited an improvement in accuracy for convenience hesitancy in the 10-shot setting (87.9%). Similarly, its *F*_1_ scores for complacency hesitancy increased from 69.3% in the zero-shot setting to 73.9% in the 10-shot setting, indicating GPT-3.5’s capacity for growth when given more examples. Llama 2, despite lower overall performance, showed consistent improvement in both accuracy and *F*_1_-scores across all hesitancy types as the number of shots increased, suggesting its potential to learn and adapt.

### Statistical Analysis for Model Comparative Analysis

We conducted a statistical comparison of GPT-4’s *F*_1_-scores across different learning paradigms for both sentiment and hesitancy analysis. In sentiment analysis, an ANOVA test indicated no significant differences (F-statistic=0.021; *P*=.981) in GPT-4’s *F*_1_-score performance across learning paradigms. Similarly, in hesitancy analysis, the ANOVA test showed no significant differences (F-statistic=0.835; *P*=.476) in GPT-4’s *F*_1_-score performance across learning paradigms. These results suggest that GPT-4’s *F*_1_-score performance remains consistent regardless of the number of training examples provided.

### Token Usage Analysis

We analyzed the number of tokens required to run the LLMs for sentiment analysis and hesitancy detection across different vaccine categories, social media platforms, and learning paradigms. Understanding token usage patterns is crucial for estimating computational costs associated with using these models. For GPT-4, the cost is US $0.03 per 1000 tokens, while for GPT-3.5, it is US $0.0005 per 1000 tokens [36]. For Claude-3 Sonnet, the cost is approximately US $0.003 per 1000 tokens [37]. Unlike these models, Llama 2 does not have a direct cost per token; instead, its cost is related to the infrastructure required to host and run the model, such as the cost of GPU servers. The cost varies based on the instance type and configuration used for deployment [38,39].

**Figure 7.**
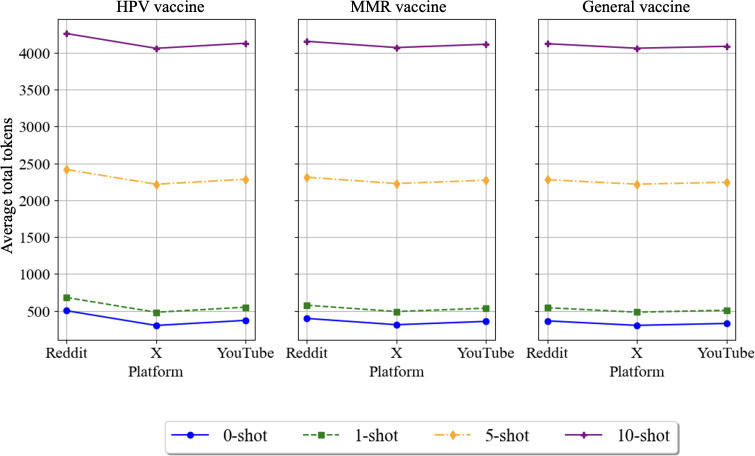
Token usage across learning paradigms and platforms for different vaccine discussions. HPV: human papillomavirus; MMR: measles, mumps, and rubella.

Analyzing the average number of tokens required per post for each combination of vaccine type, social media platform, and learning paradigm (Figure 7), the results demonstrate that the number of tokens required increases with the number of training examples provided to the models. Posts related to HPV generally require more tokens than those related to MMR and general vaccines, particularly on Reddit. This suggests that discussions surrounding HPV may be more complex or lengthy, leading to higher token usage.

Regarding social media platforms, posts from Reddit consistently require more tokens than those from YouTube and X, across all vaccine types and learning paradigms. This indicates that the longer and more detailed nature of discussions on Reddit contributes to higher token usage compared with the other platforms.

### Case-Based Evaluation of Model Performance

This section provides a case-based evaluation of misclassified posts, highlighting examples of both correct and incorrect predictions made by 4 LLMs: GPT-3.5, Llama2, Claude-3s, and GPT-4. The evaluation focuses on complex sentiments, sarcasm, and implicit support for vaccines, which often challenge LLMs in distinguishing nuanced opinions. Each post is analyzed based on its true label, the models’ predictions, and their explanations, with an assessment of where and why misclassifications occurred. To safeguard privacy, all examples presented in this section are theoretical and were crafted to reflect the types of posts analyzed in the study, rather than quoting or reproducing actual user-generated content (Textboxes 1-6).

Textbox 1. **Post analysis: explicit sentiment with mixed vaccine views.**

Post:

“You are false. The disease has a high recovery rate for healthy individuals with no underlying conditions. If I want to avoid measles, I trust the measles vaccine—it works. But the COVID-19 vaccine? It’s ineffective. Stop spreading unnecessary fear!”

- True Label: Positive (regarding MMR vaccine)
- Predictions:
  - GPT-3.5: Negative
  - Llama2: Negative
  - Claude-3s: Positive
  - GPT-4: Positive
  Explanations:
  GPT-3.5 and Llama2 focused on the aggressive tone and negative statements about the COVID-19 vaccine, overlooking the implicit endorsement of the measles vaccine. Claude-3s and GPT-4 identified the positive sentiment toward the measles vaccine despite the broader negative tone.
  Error assessment: ·
- Contextual interpretation: Models struggled with distinguishing sentiment toward different vaccines within the same post.
- Sarcasm and irony: Negative phrasing (“fear-mongering crap”) misled some models into assigning an overall negative sentiment.

Textbox 2. **Post analysis: complex sentiment with implicit support.**

Post:

“Someone compared getting vaccinated to making a noble sacrifice—seriously? Saying vaccines are pointless because only older people are at risk is both wrong and ridiculous. But whatever, it’s your choice. I’m done caring.”

- True label: Positive
- Predictions:
  - GPT-3.5: Negative
  - Llama2: Negative
  - Claude-3s: Positive
  - GPT-4: Positive

Explanations:

GPT-3.5 and Llama2 misclassified the sentiment as negative due to the critical tone and the phrase “I’m done caring,” which implies indifference. Claude-3s and GPT-4 correctly identified the critique of antivaccine reasoning as implicit support for vaccination.

Error assessment:

- Complex sentiment and implicit support: The post critiques antivaccine arguments, implicitly supporting vaccination, but uses sarcasm and frustration, which confused some models.
- Challenge in parsing ambiguity: Phrases such as “It’s your choice” were interpreted differently across models, with some seeing them as neutral or dismissive.

Textbox 3. **Post analysis: critique with ambiguous tone.**

Post:

“Oh, so being smart now means trusting pharmaceutical companies completely? Here’s the reality: those shouting about personal freedoms when asked to wear a mask seem more likely to try bizarre ‘natural remedies’ than to actually take a scientifically-proven vaccine.”

· True label: Positive
- Predictions:
  - GPT-3.5: Negative
  - Llama2: Neutral
  - Claude-3s: Negative
  - GPT-4: Neutral

Explanations:

All models struggled to classify this post correctly due to the sarcastic tone and critical phrasing. Some interpreted the critique of antivaccine rhetoric as a negative sentiment rather than implicit support for vaccination.

Error assessment:

- Complex sentiment and implicit support: The sarcastic tone and indirect language posed challenges, leading to difficulty recognizing the underlying provaccine stance.
- Misinterpretation of irony: The critique of intelligence and behavior in the context of antivaccine arguments led models to overemphasize the negative sentiment, missing the implicit support for vaccination.

Textbox 4. **Post analysis: concern over measles outbreak.**

Post:

“Measles isn’t just back in the US; we’re seeing the same issues here in Europe. It’s getting out of hand, and the number of people refusing to vaccinate their kids is shocking. %VIDEO%: The return of measles—what’s causing it?”

- True label: Positive
- Predictions:
  - GPT-3.5: Negative
  - Llama2: Neutral
  - Claude-3s: Negative
  - GPT-4: Positive

Explanations:

GPT-3.5 and Claude-3s misclassified the example due to phrases such as “out of hand” and “refusing to vaccinate,” interpreting them as indicators of negative sentiment. GPT-4 correctly identified the post as expressing concern over measles outbreaks, which implicitly supports vaccination efforts to address the issue.

Error assessment:

- Complexity in contextual signals: The use of emotionally charged phrases such as “out of hand” misled some models into perceiving the sentiment as negative, despite the post’s implicit advocacy for vaccination to control outbreaks.

Textbox 5. **Post analysis: sarcastic critique of herd immunity arguments.**

Post:

“Be sure to tell everyone you’re skipping vaccines for diseases like polio, rubella, or whooping cough. Herd immunity will handle it, right? A few thousand dead kids is just the cost of freedom. Moo!”

- True label: Positive
- Predictions:
  - GPT-3.5: Negative
  - Llama2: Negative
  - Claude-3s: Negative
  - GPT-4: Positive

Explanations:

GPT-3.5, Llama2, and Claude-3s struggled with interpreting the sarcastic tone, focusing on phrases such as “dead kids” as indicators of negative sentiment. GPT-4 correctly understood the sarcasm as a critique of antivaccine reasoning, identifying the post’s implicit support for vaccination.

Error assessment:

- Failure to recognize sarcasm: Most models struggled with the heavily sarcastic tone, focusing on surface-level negative phrases rather than the implicit provaccine stance.

Textbox 6. **Post analysis for constructs.**

Post:

“I thought about getting the HPV vaccine, but then I found out it doesn’t cover all strains. What’s the point if I could still catch something? Plus, the nearest clinic is too far, and I can’t miss work just for a shot. Maybe I’ll wait and see.”

- True label: Hesitant (Confidence + Convenience)
- Predictions:
  - GPT-4: Hesitant (confidence)
  - Claude-3s: Hesitant (complacency)
  - GPT-3.5: Nonhesitant
  - Llama2: Nonhesitant

Explanations:

GPT-4 successfully identified the confidence issues (concerns about the vaccine’s incomplete coverage) but overlooked the logistical barrier related to clinic distance and work constraints. Claude-3s misclassified the post as complacency, interpreting “wait and see” as indicating low-perceived disease risk, rather than logistical or confidence-related barriers. GPT-3.5 and Llama2 both classified the post as nonhesitant, failing to recognize the implicit skepticism and practical challenges expressed in the post.

Error assessment:

- Difficulty in addressing multiconstruct hesitancy: The intertwined nature of confidence (concerns about vaccine efficacy) and convenience (logistical challenges) constructs posed challenges for all models.
- Oversimplification of constructs: Models such as Claude-3s and GPT-4 overemphasized individual constructs, leading to incomplete classification.
- Missed implicit barriers: GPT-3.5 and Llama2 failed to interpret the implicit logistical challenges as indicative of hesitancy, instead categorizing the post as nonhesitant.

### Arguments

This case-based evaluation highlights the challenges faced by LLMs in accurately classifying nuanced and complex sentiments. Models often misclassify posts due to sarcasm, indirect language, or the presence of mixed sentiments targeting different aspects of vaccination. While GPT-4 demonstrated the highest accuracy in identifying implicit support, other models frequently struggled with contextual interpretation. Future work could focus on improving models’ ability to handle sarcasm, irony, and subtle expressions of support or critique.

## Discussion

### Principal Findings

This study evaluated the effectiveness of LLMs in sentiment analysis and hesitancy detection regarding vaccine-related posts on social media, focusing on models including GPT-3.5, GPT-4, Claude-3 Sonnet, and Llama 2. Our results indicate that GPT-4 outperforms other models across various metrics, consistent with recent advancements in LLM capabilities for processing complex linguistic patterns [40].

### Sentiment and Hesitancy Analysis Insights

GPT-4 consistently outperformed other models across all sentiment types and learning paradigms, attributable to its larger parameter size and extensive pretraining on diverse datasets, enabling it to capture subtle nuances and contextual information more effectively [41]. However, our analysis also revealed that the performance of smaller LLMs, such as Claude 3s and GPT-3.5, was not far behind GPT-4, particularly in the few-shot learning paradigm. Although fine-tuning was not conducted in this study, previous research has shown that fine-tuned smaller LLMs can achieve performance levels similar to state-of-the-art models in some domain-specific applications, such as financial sentiment analysis [41]. Exploring fine-tuning as a future direction could further enhance these models’ task-specific performance. From a cost perspective, smaller models can be a more viable and budget-friendly alternative. For instance, GPT-3.5 costs approximately US $0.0005 per 1000 tokens, while GPT-4 costs around US $0.03 per 1000 tokens [36]. This substantial difference in cost highlights the economic advantage of using smaller LLMs for large-scale applications where budget constraints are a factor. Moreover, the slightly reduced performance of smaller LLMs in comparison with larger models might be offset by their significantly lower operational costs, making them a practical choice for many real-world applications.

Our study found that GPT-4 handles discussions about HPV vaccines better than those about general vaccines or MMR, likely due to the more consistent language used in HPV discussions. In contrast, conversations about general vaccines or MMR cover a broader range of topics, making it harder for models to perform consistently well. In addition, the platform impacts model performance, with Reddit allowing for longer, more complex discussions, and YouTube comments often using informal language and slang.

The performance of LLMs in classifying neutral sentiments revealed notable challenges, highlighting the inherent difficulties due to their less expressive nature and greater reliance on contextual understanding. Enhanced model training that better captures the subtleties of neutral language and incorporates contextual cues is necessary for improving neutral sentiment classification.

In hesitancy analysis, GPT-4 and Claude-3 Sonnet led the pack. Interestingly, the models generally achieved higher accuracy in hesitancy analysis than in sentiment analysis, likely due to the more specific nature of hesitancy categories. However, lower *F*_1_-scores for the complacency hesitancy type indicate challenges in capturing nuanced and context-dependent expressions of hesitancy. Complacency, often expressed through passive language that neither outright rejects nor endorses vaccination, requires a nuanced detection approach that current models struggle to provide. This issue highlights the need for improvements in model training, suggesting that integrating more complex, contextually rich datasets could enhance model sensitivity to the passive expressions typical of complacency.

### Consistency Across Learning Paradigms

Our analysis revealed that increasing the number of shots did not always lead to improved performance, aligning with findings that few-shot learning’s effectiveness depends on the quality and relevance of the examples provided [41]. GPT-4 showed consistent performance across zero-shot, 1-shot, and few-shot learning paradigms, with no significant differences in performance enhancements with more training examples. This consistency makes zero-shot learning a cost-effective strategy due to its balance of accuracy and reduced computational resources [42,43].

Our token usage analysis indicated that the number of tokens required increased with the number of training examples, with the zero-shot paradigm requiring the least tokens and the 10-shot paradigm requiring the most. Token usage also varied across vaccine types and social media platforms, suggesting that the complexity and length of discussions impact computational costs.

While LLMs such as GPT-4 offer superior performance metrics, these benefits come with higher computational expenses and longer processing times. For instance, GPT-4 achieved an accuracy of 88% and an *F*_1_-score of 0.85, outperforming traditional machine learning methods [23]. However, the associated usage costs and processing times pose challenges for large-scale, real-time deployment.

### Hybrid Approach for Improved Efficiency

To address these challenges, we propose a hybrid approach leveraging both LLMs and traditional machine learning methods, combined with manual annotation. LLMs such as GPT-4 can handle a significant portion of the annotation workload, processing large volumes of data efficiently and providing consistent, high-quality annotations based on predefined criteria, reducing the burden of manual annotation and minimizing variability associated with human annotators.

A subset of the data should undergo manual annotation by human experts to ensure that nuanced and complex cases are accurately labeled, providing a benchmark to validate and refine LLM-generated annotations. Once annotated, traditional machine learning models can be trained using this hybrid-annotated dataset. These models, being more computationally efficient, can be deployed for real-time monitoring and analysis of social media data.

By integrating automated and manual annotation methods, we optimize both performance and cost, ensuring accurate and timely insights into vaccine sentiment and hesitancy. This approach enhances the feasibility of using advanced NLP techniques in public health and sets a precedent for future research in leveraging hybrid models for complex analytical tasks.

### Insights From Case-Based Evaluation

The case-based evaluation underscores the complexities faced by LLMs when classifying nuanced sentiments and vaccine hesitancy constructs in social media posts. Posts containing sarcasm, indirect phrasing, and implicit sentiments were particularly challenging for models. For instance, sarcastic critiques of antivaccine rhetoric were frequently misclassified as negative sentiment, highlighting the limitations of LLMs in interpreting figurative language and irony. This aligns with previous findings that sarcasm and indirect expressions pose significant challenges in text classification tasks [44].

GPT-4 consistently exhibited better contextual understanding, particularly in recognizing implicit support for vaccination. However, even GPT-4 struggled with multiconstruct hesitancy posts, such as those intertwining confidence issues and logistical barriers. Other models, such as GPT-3.5, Llama2, and Claude-3s, often misclassified posts by overrelying on surface-level cues, failing to capture the deeper sentiment or hesitancy constructs. These observations reflect broader limitations in LLMs’ ability to handle multidimensional health-related discourse.

The evaluation highlights the need for improved training and fine-tuning strategies that incorporate domain-specific linguistic features and multilabel constructs. Future research should focus on enhancing models’ ability to parse complex sentiment, handle mixed tone, and interpret multiconstruct hesitancy more effectively.

### Limitations

#### Dataset Size and Representation

A key limitation of this study is the size of the evaluation dataset, comprising 7515 annotated social media posts. While efforts were made to ensure balance across sentiment and hesitancy categories, certain subcategories, such as complacency and convenience within hesitancy, may still contain relatively small counts. This could limit the robustness of conclusions drawn for these specific subcategories. Furthermore, the reuse of the same training posts across few-shot learning experiments was a deliberate choice to maintain consistency and enable a controlled comparison of model performance. However, this may limit the diversity of training examples, which could impact the generalizability of the findings. Future studies could address these limitations by expanding the dataset, particularly within less-represented categories, and exploring the effects of more diverse training samples on LLM performance.

#### Contextual Influences and Pandemic Polarization

The data used in this study span up until October 2021, encompassing the period of the COVID-19 pandemic. The COVID-19 pandemic created a highly polarized environment around vaccine discussions on social media, amplifying both supportive and skeptical voices. This polarization likely influenced the data composition, with heightened expressions of hesitancy driven by safety concerns, misinformation, and politicized discourse. These patterns may have shaped the ability of LLMs to classify sentiments and hesitancy constructs, particularly when handling mixed tones or ambiguous expressions. For instance, the increased prevalence of emotionally charged or politicized language during the pandemic might have led to overemphasis on negative cues in posts that were otherwise supportive of vaccination. Addressing these nuances requires models capable of disentangling context-dependent sentiments, a challenge underscored in this study’s findings. Future research could investigate how temporal factors, such as the pandemic’s progression, impact sentiment and hesitancy patterns.

#### Limitations of API-Based Models

While our study offers valuable insights into the performance of LLMs in analyzing vaccine-related sentiments and hesitancy on social media, it is important to acknowledge several limitations that future research should address. First, our analysis was limited to the models available through Azure and Amazon Bedrock’s API services at the time of our research. These included GPT-3.5, GPT-4, Claude-3 Sonnet, and Llama 2. While these models were accessible and suitable, given our computational resources, they were not necessarily the most advanced versions available. The rapid development in the field of LLMs means that more recent and potentially more effective models could offer enhanced performance on similar tasks. Also, the reliance on API versions available through Azure and Amazon Web Services means that the models and tools we used may not have been the most optimized for our specific research purposes. API versions are frequently updated to include improvements and new features that could significantly enhance model performance. Consequently, subsequent research should strive to use the most recent API versions to ensure that findings are based on the best possible technological tools and to maintain comparability with other contemporary research.

#### Limited Vaccine Types and Platforms

Our analysis focused on a limited set of vaccine types and social media platforms. While these choices were based on data availability and relevance to our research questions, they may not capture the full spectrum of vaccine-related discussions on social media. Future research could expand the scope to include a wider range of vaccine types and platforms to assess the generalizability of our findings and identify potential differences in sentiment and hesitancy patterns across various contexts.

### Practical Integration and Ethical Considerations

This study focused on the technical aspects of using LLMs for vaccine sentiment analysis and hesitancy detection, but it did not delve into the practical implications and challenges of integrating these models into real-world public health initiatives. Future research should explore the ethical, social, and organizational factors that may influence the adoption and effectiveness of LLMs in addressing vaccine hesitancy. This could include investigating issues such as data privacy, algorithmic fairness, and the potential unintended consequences of using LLMs in public health communication strategies.

While our study highlights the potential of LLMs in identifying trends in vaccine hesitancy, it is important to recognize that these models are just one piece of the puzzle. Combating vaccine hesitancy requires a multifaceted approach that combines data-driven insights with effective communication strategies, community engagement, and trust-building efforts. Future research should explore how LLMs can be integrated into comprehensive public health interventions that address the complex and multidimensional nature of vaccine hesitancy.

### Future Directions

#### Advanced Prompting Techniques

Future work could explore innovative prompting methods such as chain of thought reasoning and clue and reasoning prompting. These approaches could enhance the reasoning capabilities of LLMs, particularly in tasks requiring interpretation of sarcasm, indirect language, or multiconstruct hesitancy. Additionally, retrieval-augmented generation could be used to retrieve contextually relevant examples for improved performance during inference.

#### Domain-Specific Fine-Tuning

While fine-tuning was not conducted in this study, its potential to improve performance in domain-specific tasks warrants exploration. For instance, fine-tuning smaller models such as GPT-3.5 on datasets with domain-specific linguistic features or domain-specific datasets enriched with figurative language, sarcasm, and implicit expressions could enhance their ability to capture vaccine-related nuances while maintaining cost efficiency.

#### Dataset Expansion and Diversification

Increasing the size and diversity of datasets, particularly within underrepresented categories such as complacency and convenience, could provide more robust training and evaluation data. In addition, temporal analyses comparing pre- and postpandemic datasets could help identify shifts in public sentiment and hesitancy patterns.

#### Cross-Platform and Vaccine Type Analyses

Expanding the study to include additional social media platforms and vaccine types could improve generalizability and provide deeper insights into context-specific discourse patterns. Platforms such as Instagram or TikTok, which cater to different user demographics, may reveal distinct sentiment trends.

#### Ethical and Practical Considerations

Future research should explore the ethical implications of deploying LLMs in public health communication. This includes addressing algorithmic biases, ensuring data privacy, and minimizing unintended consequences, such as the spread of misinformation.

#### Development of Novel Metrics

Traditional metrics, such as *F*_1_-scores, are insufficient for capturing performance in nuanced tasks such as sarcasm detection and implicit sentiment classification. Future studies should develop novel metrics, such as a “sarcasm sensitivity index” or an “implicit sentiment alignment score,” to better evaluate LLMs in these complex areas.

#### Explainability and Interpretability Research

Investigating explainability methods to understand LLMs’ decision-making processes is a promising direction. For example, using attention heatmaps to visualize how models focus on specific words or phrases could reveal their interpretation mechanisms. This could help refine models to better handle mixed or ambiguous language constructs, such as multiconstruct hesitancy posts.

### Conclusions

Our study provides a foundation for understanding the performance and limitations of LLMs in vaccine sentiment analysis and hesitancy detection tasks. However, there are several limitations and opportunities for future research that should be addressed to fully harness the potential of these models in promoting vaccine acceptance and uptake. As the field of LLMs continues to evolve rapidly, researchers and public health professionals should remain vigilant in evaluating the latest developments and adapting their approaches to ensure the most effective and responsible use of these powerful tools in addressing the critical challenge of vaccine hesitancy.

## Supplementary material

10.2196/64723 Multimedia Appendix 1 Number of annotated posts for each social media platform, categorized by vaccine sentiment and World Health Organization’s 3Cs (confidence, complacency, and convenience) model vaccine hesitancy group. HPV: human papillomavirus vaccines; MMR: measles, mumps, and rubella vaccines; General: general or unspecified vaccines.

10.2196/64723 Multimedia Appendix 2 Prompt schema.

## Abbreviations

AI: artificial intelligence
API: application programming interface
GPT: generative pretrained transformer
HPV: human papillomavirus
LLM: large language model
MMR: measles, mumps, and rubella
NLP: natural language processing
3Cs: confidence, complacency, and convenience
WHO: World Health Organization
