# Supplementary material for: Evaluating Large Language Models for Sentiment Analysis and Hesitancy Analysis on Vaccine Posts From Social Media: Qualitative Study
Source: JMIR Form Res. 2025 Oct 15;9:e64723. doi: 10.2196/64723 (PMC12526656; doi:10.2196/64723)
Supplement: Multimedia Appendix 1 [file formative-v9-e64723-s001.docx]

Multimedia Appendix 1

Table S1. Number of annotated posts for each social media platform, categorized by vaccine sentiment and World Health Organization’s 3Cs model vaccine hesitancy group. HPV: human papillomavirus vaccines; MMR: measles, mumps, and rubella vaccines; General: general/unspecified vaccines.

| **Dataset** | | | **Sentiment** | | | **WHO’s 3Cs Models** | | | | | | | | **Hesitancy** | | |  |
| --- | --- | --- | --- | --- | --- | --- | --- | --- | --- | --- | --- | --- | --- | --- | --- | --- | --- |
|  |  |  |  |  |  | **Confidence** | | **Complacency** | | | **Convenience** | | |  |  |  |  |
| **Platform** | **Vaccine topic** | **# of posts** | **Positive** | **Neutral** | **Negative** | **Confident** | **Lack of confidence** | | **Complacent** | **No complacency** | | **Convenient** | **Inconvenient** | | **Hesitant** | **Nonhesitant** | |
| Twitter | HPV | 1165 | 683 | 365 | 117 | 85 | 195 | | 42 | 203 | | 214 | 18 | | 232 | 933 | |
|  | MMR | 1165 | 478 | 780 | 297 | 55 | 520 | | 52 | 535 | | 582 | 6 | | 588 | 1045 | |
|  | General | 1165 | 182 | 796 | 187 | 110 | 218 | | 101 | 229 | | 320 | 10 | | 330 | 835 | |
| Reddit | HPV | 1165 | 484 | 545 | 136 | 14 | 62 | | 5 | 71 | | 67 | 10 | | 77 | 1088 | |
|  | MMR | 1165 | 394 | 593 | 178 | 63 | 72 | | 61 | 74 | | 132 | 2 | | 134 | 1031 | |
|  | General | 1165 | 170 | 910 | 85 | 13 | 57 | | 7 | 62 | | 64 | 5 | | 69 | 1096 | |
| YouTube | HPV | 1165 | 334 | 467 | 364 | 66 | 293 | | 57 | 303 | | 351 | 10 | | 361 | 804 | |
|  | MMR | 1165 | 365 | 627 | 173 | 7 | 311 | | 5 | 316 | | 318 | 3 | | 321 | 844 | |
|  | General | 1165 | 109 | 579 | 477 | 19 | 412 | | 19 | 412 | | 429 | 3 | | 432 | 733 | |
