# Supplementary material for: Evaluating Large Language Models for Sentiment Analysis and Hesitancy Analysis on Vaccine Posts From Social Media: Qualitative Study
Source: JMIR Form Res. 2025 Oct 15;9:e64723. doi: 10.2196/64723 (PMC12526656; doi:10.2196/64723)
Supplement: Multimedia Appendix 2 [file formative-v9-e64723-s002.docx]

Multimedia Appendix 2

**Prompt Schema for Sentiment Analysis Tasks**

The following schemas were used to construct prompts for evaluating sentiment towards vaccines across different contexts and learning paradigms. Each prompt includes definitions for sentiment categories, and specific instructions

**Zero-Shot Prompt Schema for MMR Vaccine:**

You are given a post related to vaccination, specifically mentioning the MMR vaccine. Your task is to determine the sentiment of the tweet towards the MMR vaccine excluding any comparisons or discussions related to other vaccines. Consider only the segment that directly discusses the MMR vaccine for your analysis. Use the following definitions strictly:

Positive: The tweet shows positive opinions on the MMR vaccine, implies criticism of not getting the HPV vaccine, or mentions news or stories that are helpful for the promotion of HPV vaccination.

Negative: The tweet shows negative opinions on the MMR vaccine or expresses negative opinions towards getting vaccinated against MMR.

Neutral: The tweet is related to the MMR vaccine and vaccination topics but contains no sentiment or the sentiment is unclear or contains both negative and positive sentiment without a clear stance on the MMR vaccine.

If input contains Origin and Quote segments, return sentiment of Quote segment ONLY.

Else If input contains COMMENT, ORIGIN, or VIDEO segments, return sentiment of COMMENT segment ONLY.

Format your response as JSON object with “Sentiment” and "Explanation" as keys.

Post: '''{text}'''

**Prompt Schema for Hesitancy Analysis:**

**Zero-Shot Prompt Schema for General Vaccine:**
You are analyzing a social media post about vaccination. Your task is to determine if the post demonstrates vaccine hesitancy. If hesitancy is detected, classify the post using the WHO's 3Cs model of vaccine hesitancy: Confidence, Complacency, and Convenience. Guidelines for classification:

- "Confidence": Distrust in vaccine safety, efficacy, policymakers, or health authorities. - "Complacency": Belief that vaccination is unnecessary due to low disease risk. - "Convenience": Barriers to vaccination, such as availability, accessibility, cost, or time. Important Instructions:

1. Posts may exhibit one, multiple, or none of these hesitancy categories.

2. If the post supports vaccination or demonstrates no hesitancy, classify it as "Non-Hesitant."

3. For ambiguous cases or posts reflecting overlapping constructs, prioritize the construct most explicitly expressed.

4. If the post is unrelated to vaccines, classify it as "Irrelevant."

Formatting:

Respond in JSON format with:

- "Hesitancy": ("Hesitant" or "Non-Hesitant")

- "Categories": (Include applicable 3Cs categories or leave empty for Non-Hesitant posts)

-"Explanation": (Brief reasoning for your classification)

Analyze the following post and classify it: Post: '''{text}'''
